# Distributed computing for the reconstruction of multi-terabyte tomographic X-ray imaging datasets

**DOI:** 10.1107/S1600577526004856

**Published:** 2026-06-02

**Authors:** Thorbjørn Erik Køppen Christensen, Frederik Holm Gjørup, Mads Ry Vogel Jørgensen, Adrian Rodriguez-Palomo, Anders Bjorholm Dahl, Innokenty Kantor

**Affiliations:** a DTU Compute, DTU 324, Kongens Lyngby, Denmark; b MAX IV Laboratory Lund University Fotongatan 2 Lund Sweden; c Department of Chemistry and iNANO Aarhus University Langelandsgade 140 Aarhus Denmark; d DTU Physics, Fysikvej 10, Kongens Lyngby, Denmark; Australian Synchrotron, Australia

**Keywords:** big data, tomography, reconstruction, materials science

## Abstract

Tomographic datasets have continued to grow in size and volume along with camera technology and synchrotron brilliance. These larger datasets require new methods of distributing the reconstruction process on high-performance computing systems. Here we present a program for reconstructing large datasets, allowing the usage of either CPU- or GPU-powered clusters, and show the successful reconstruction of a 24000 pixel-wide dataset. This program is currently in use at the DanMAX beamline at the MAX IV synchrotron and is used as the default reconstruction method for all tomographic experiments at the beamline. Scans reconstructed with the presented pipeline have been published in multiple journals.

## Abstract 2

As tomographic datasets from synchrotrons continue to increase in volume, so rises the challenge of reconstructing said data. Here we present a solution utilizing high-performance computing capabilities to split the process into multiple smaller tasks, which can be solved without overwhelming a single compute node. This solution is currently in use at the DanMAX beamline at the MAX IV synchrotron.

## 1. Introduction

Tomographic full-field imaging is an essential technique for studies of materials, medicine, archaeology and biology alike, as it allows for non-destructive examination of the internal microstructure of materials in 3D. A fundamental part of tomographic imaging is the reconstruction process. Tomographic reconstruction is well established; however, it can be a bottleneck due to the large data volumes. The reconstruction process can typically be split into three major parts: corrections and pre-processing, the reconstruction itself, and post-processing.

There are multiple different corrections and preparatory computations to apply, depending on how the data are measured. For a parallel beam setup, such as the ones typically found at synchrotrons, the most typical are flat- and dark-field corrections, and phase retrieval (Paganin *et al.*, 2002 ▸; Paganin & Pelliccia, 2021 ▸; Gürsoy *et al.*, 2014 ▸). After the images have been pre-processed, the data can be reconstructed using one of the many reconstruction algorithms available. The most common algorithms are filtered back-projection (Radon, 1986 ▸) and gridding reconstruction (gridrec) (Marone & Stampanoni, 2012 ▸). The post-processing step often includes dynamic range compression (*e.g.* from 32-bit to 16- or even 8-bit dynamic range), ring artefacts removal, circular mask application, *etc*. These reconstruction algorithms, along with the pre- and post-processing algorithms, are implemented in libraries to use for the reconstruction of datasets, such as *TomoPy* (Gürsoy *et al.*, 2014 ▸), *TomocuPy* (Nikitin, 2023 ▸) and *ASTRA* (van Aarle *et al.*, 2015 ▸). These libraries are all highly versatile and cater to slightly different use cases.

The above-mentioned frameworks are remarkably efficient in reconstructing datasets. However, with new detectors and fourth-generation synchrotrons (Tavares *et al.*, 2014 ▸; Tavares *et al.*, 2018 ▸; Liu *et al.*, 2019 ▸; Raimondi, 2016 ▸; Raimondi *et al.*, 2023 ▸), the speed and volume of the acquired data increase rapidly, requiring a framework for distributing this workload across multiple machines in high-performance computing (HPC) centres. Here we present such a framework, utilizing the *TomoPy* (Gürsoy *et al.*, 2014 ▸) and *TomocuPy* (Nikitin, 2023 ▸) libraries for the underlying calculations, but allowing for the reconstruction of much larger datasets than otherwise possible. This is done by splitting the computation into two to four passes. This does require writing and loading the data to disk, which slows down the overall process but allows for larger datasets.

## 2. Materials and methods

We begin by providing an overview of the data pathway, followed by descriptions of experimental data that require this scheme for reconstruction.

### 2.1. Reconstruction on an HPC scheme

**Figure 1:**
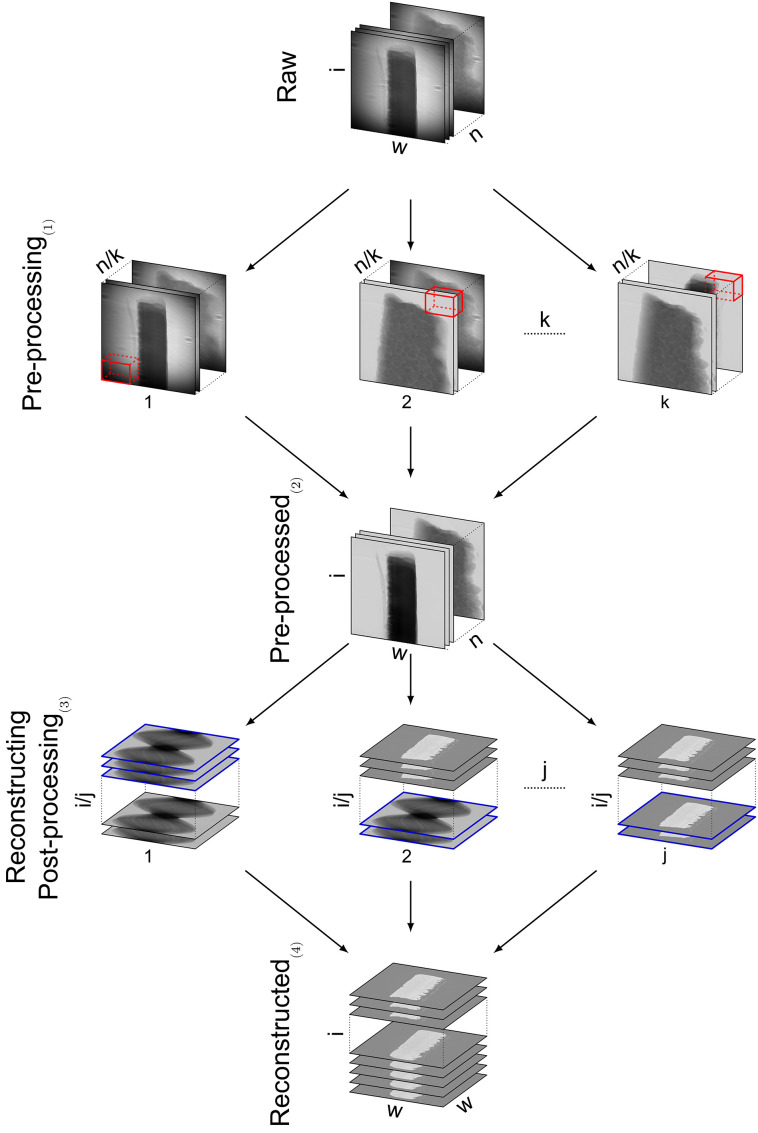
Data processing scheme, showing how data are split and collected. In the beginning the raw data are split into multiple jobs to be pre-processed in chunks, each chunk is then pre-processed in smaller subchunks (marked in red). The pre-processed data are then collected virtually, before being split again for the reconstruction and post-processing, again in a chunk/subchunk scheme (*j* chunks with subchunks marked in blue). Lastly the data can be physically collected. The sample is a piece of eggshell with both shell and membrane visible. The numbers on the figure correspond to the numbers of the corresponding steps in the description.

An overview of the process used for reconstruction on an HPC system can be seen in Fig. 1 ▸. The process of reconstruction can be split into three major parts: pre-processing, reconstruction and post-processing. The implementation of the framework requires that the raw data are stored in the DXchange format (De Carlo *et al.*, 2014 ▸), as this allows the reconstruction with only little information besides that found in the file. In particular, the pipeline requires information on the centre of rotation value and the sample properties (provided as a ratio between the real and the imaginary parts of its refractive index) required for the phase retrieval (Paganin *et al.*, 2002 ▸). The program, which acts as a SLURM (Yoo *et al.*, 2003 ▸) interface, assumes that settings for the reconstruction are provided by the user, but automatically takes care of spawning and terminating jobs. The overall process can be described as follows, assuming a dataset with *n* projections and *i* slices and a sensor size with a width of *w* pixels:

(1) Split the data into *k* chunks of *n*/*k* projections. Every chunk will have a job in the HPC queueing system. This implementation is for SLURM.

 (*a*) Split every chunk into subchunks. The subchunks should be able to fit in memory and should adhere to some limitations depending on the requirements of the wanted pre-process steps.

 (*b*) Add to every subchunk an overlap region with the neighbouring subchunk, ensuring that pre-processing, *e.g.* phase retrieval or lens distortion corrections, which require neighbouring pixels, can be applied. The size of the overlap depends on the magnitude of the neighbour-dependent corrections. For every subchunk:

  (I) Load the subchunk data.

  (II) Apply pre-processing and corrections: normalize using white- and dark-field images; apply *I*_0_ correction to correct for top-ups during the scan; outlier removal; stripe removal; air correction; retrieve phase using the Paganin method (Paganin *et al.*, 2002 ▸; Paganin & Pelliccia, 2021 ▸). The corrections can be switched on and off in the configuration, and more can easily be added should it be deemed necessary

  (III) Write the subchunk data, excluding the overlap region, to one file for every one of the *k* data chunks, avoiding IO conflicts. Go to step (I) if there are more subchunks in the data chunk.

(2) Virtually combine the *k* pre-processed data files using HDF5 virtual datasets to create an intermediate DXchange file without the overhead associated with reading any data.

(3) Split the data into *j* chunks of *i/j* sinograms each. Every chunk will have a job on the queueing system:

 (*a*) Split each chunk into subchunks with a number of sinograms that can be fully reconstructed in memory at the same time.

 (*b*) Load the subchunk.

 (*c*) Apply the sinogram-dependant pre-process steps: apply −log to the data to get absorption; if using the ‘half-acquisition’ scheme (when the centre of rotation is close to the edge of a projection, and a full rotation is measured) convert the data from 360° to 180; apply padding. Finally, apply the stripe removal.

 (*d*) Run the reconstruction on the subchunk.

 (*e*) If required: run ring removal.

 (*f*) If required: apply circular mask.

 (*g*) If required: convert the data to a different format/bit depth.

 (*h*) Write the subchunk to the *j*th data file, with one file for every chunk, avoiding IO conflicts. Go to step (*b*) if more subchunks are left unprocessed.

(4) Combine the reconstructed files into a physical file or create a virtual collection of the reconstructed files. If required, down-sample the data to create an hierarchical data structure.

(5) Create a reconstruction report for the reconstruction process, showing orthogonal slices of the raw data, corrected data and reconstructed data, along with information about the reconstruction in a PDF file.

(6) Remove all extraneous files, such as job files, from the queueing system and intermediary data files.

While the approach of reading and writing the data multiple times has an additional cost of IO time, it allows for the subchunks to be optimized for their respective purposes: for phase retrieval, multiple lines from the projection *must* be present, as the phase fringes are much larger than one pixel. This is especially important at a beamline such as DanMAX, where the rotation axis is parallel to the axis of highest coherence, meaning that the fringes are stronger orthogonal to the tomographic slice than within the slice, and the phase retrieval is thus more important orthogonal to the slices. This is the opposite of what is needed for the reconstruction, where the full width *and* depth are necessary. The full width is necessary for some corrections, such as the *TomoPy* air correction, which assumes air on both sides of the sample, and the air pixel values are used to compensate for any intensity or flat-field instabilities. In such cases, the subchunk must have the full width of the chunk. For stripe removal (Vo *et al.*, 2018 ▸; Münch *et al.*, 2009 ▸; Miqueles *et al.*, 2014 ▸), it is necessary to have depth in the picture; however, this can be applied if only a subset of angles are present, so the full sinogram is not necessary. As having a full dataset loaded is often not possible, this subchunking allows the data to be loaded in a fashion that makes all pre-processing steps possible.

**Figure 2:**
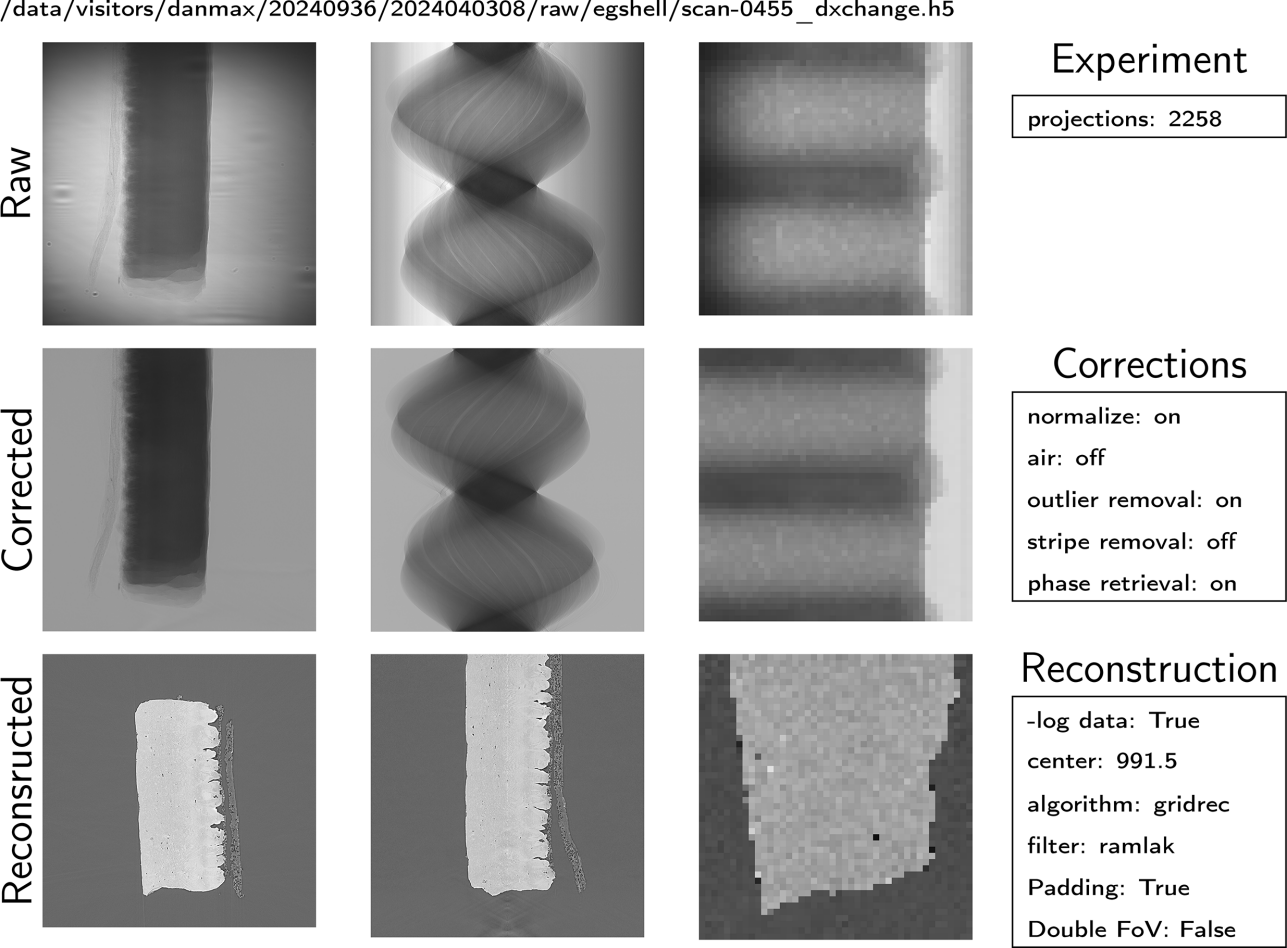
Example of the summary saved as a PDF for showing the reconstruction process generated by the framework. The raw, corrected and reconstructed data with three orthonormal slices are shown from each dataset. The report indicates which corrections were applied, and which reconstruction method was used. Note that the third image is deliberately shown in low resolution, as reading in this direction is computationally expensive. It is customizable in the pipeline how high the sampling of these slices should be. The sample in the example is a piece of chicken egg shell, with both membrane and shell visible.

A summary PDF file produced by the pipeline enables the user to quickly check whether the dataset has been reconstructed properly, without needing to load the dataset in a file viewer. It also makes it possible to check whether any errors happened in the correction or reconstruction step. An example of a reconstruction summary PDF file is shown in Fig. 2 ▸. The summary shows which file was reconstructed, a raw projection and sinogram, a corrected projection and sinogram, and three orthogonal slices from the reconstructed file. Furthermore, experimental and processing information are shown.

Storing the intermediate files would at least double the long-term storage need for the data, so the intermediary data are deleted along with the job-files created by the SLURM system, after the PDF file has been created.

### 2.2. Scanning methods

**Figure 3:**
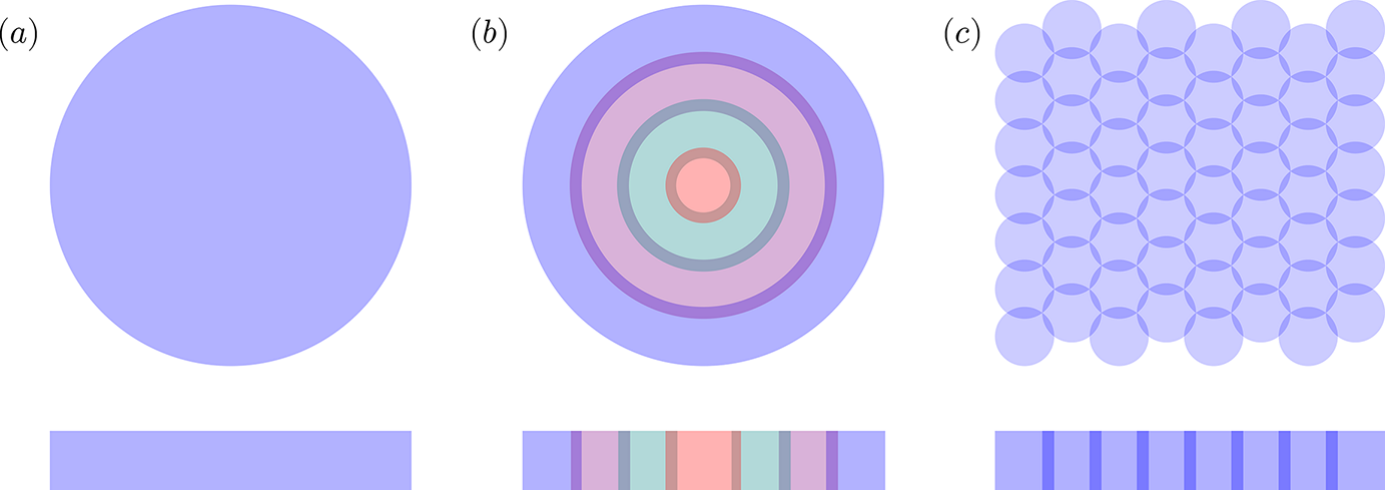
Different measurement strategies for high-resolution and large field of view datasets. The top view shows a cross-sectional overview of the scanned volume. The bottom view is perpendicular to the top view. (*a*) Wide beam with a large camera sensor with a high pixel count. (*b*) Projection stitching, multiple scans with projections stitched together pre-reconstruction to create a full field tomogram. Each colour represents two scans, one on the left and right, for a total of seven 180° scans or four 360° scans. The overlap is needed to align projections. (*c*) Volume stitching – multiple local tomograms stitched together volumetrically post-reconstruction.

While this pipeline can be and currently is used for bulk reconstruction of single scans at the DanMAX beamline, the main purpose of this development is the reconstruction of extremely large tomograms. There are three major ways to measure tomograms with a high pixel count across the projections; these are shown in Fig. 3 ▸. The ideal and simplest option is using a detector with a big sensor combined with a large beam (Yakovlev *et al.*, 2022 ▸); this is shown in Fig. 3 ▸(*a*). The DanMAX beam has a size of approximately 1.3 mm × 1.3 mm, and without specialty hardware, such as additional X-ray optics (Boettinger *et al.*, 1979 ▸; Snigirev *et al.*, 1998 ▸; Kubec *et al.*, 2017 ▸) and high-pixel-count cameras (Yakovlev *et al.*, 2022 ▸), it is not possible to scan large areas using a large beam. While not utilized here, there is ongoing work to install beam-expanders and a high-pixel-count detector at the DanMAX beamline (Gellert *et al.*, 2025 ▸).

Another option is to scan multiple sets of projections, and then combine them prior to the reconstruction, to form large full-field projections (Vescovi *et al.*, 2018 ▸). As shown in Fig. 3 ▸(*b*), this requires a small overlap between the projections. It is necessary to collect enough projections in each position to match the combined number of pixels across the combined projections. This entails long scanning times at multiple positions. Scanning the full projection as shown in Fig. 3 ▸(*b*) requires seven separate 180° scans or four separate 360° scans in total. If the sample changes between these scans, for example, due to beam damage, the reconstruction will likely be compromised by artefacts. The rotation centre is the same for all scans.

The last way is to measure multiple small local tomograms, each with their own rotation centre, as seen in Fig. 3 ▸(*c*). These volumes can then be stitched together volumetrically after reconstructing (Vescovi *et al.*, 2018 ▸). The fast scanning time for each local tomogram means that the sample has a smaller chance of changing during the scan. However, there is an increased chance of local tomography artefacts. It is thus preferable for more uniform samples. This method allows the creation of high-pixel-count tomograms, but does not test the reconstruction of large datasets, and hence will not be used here.

Here we proceed with data collected using projection stitching methods, as shown in Fig. 3 ▸(*b*).

### 2.3. Projection stitching method

To stitch projections, the position of the scans was first found based on the first projection of each rotation scan. The motor position was then used as an initial guess, around which an area of 20×20 pixels was tested, and the minimum difference between the two images in that was then used to correct for inaccuracy in the motors, as the pixel size of the scans is 275 nm, which is finer than the motor precision. To gradually blend the images on stitching, the distance from the edge is used to calculate relative weights for the overlapping image regions, further normalized to 1 between the overlapping images. Other options for stitching images are available, such as FFT-based methods (Guizar-Sicairos *et al.*, 2008 ▸), but were not tested here.

### 2.4. Samples

Three samples were used for testing, one sample yielding primarily phase contrast, and two samples dominated by the absorption contrast. For the phase object, half a mouse brain was used. The mouse brain was embedded in paraffin (not depicted).

The first absorption sample was a piece of ovine bone sourced from a local butcher. The sample was cut to a diameter of 4 mm using a micro-lathe (Holler *et al.*, 2020 ▸; Wittig *et al.*, 2024 ▸), but was free-standing during the scanning. The second was a piece of chicken eggshell, which had been boiled prior to scanning.

### 2.5. Experimental

All scans were recorded using a 12 Mpix sCMOS camera (Hamamatsu Orca Lightning C14120, 5.5 µm × 5.5 µm pixel size and a resolution of 4608 × 2592 pixels), with an energy of 20 keV and a flux of 10^12^ photons s^−1^. Note that, for the scans using the 20× objective, half the beam is slit away, as the beam covers the full width of the detector but twice the height, thus approximately halving the flux when running with the 20× objective.

The brain and bone samples were scanned with an X-ray microscope based on a 20× long-range infinity corrected objective from Mitutoyo and a 50 µm-thick Lu Ce-doped lutetium aluminium garnet scintillator in a ‘white beam’ geometry.

The brain was scanned with an exposure time of 15 ms and latency of 33 ms per projection and 24002 projections total. The sample-to-detector distance was 3.0 cm. Six scans were necessary to cover the full width of the sample. The resulting volume was of size 2586 × 24608 × 24608 voxels, corresponding to a data volume of 5.7 TiB.

The bone was scanned with an exposure time of 15 ms and latency of 33 ms per projection and 25134 projections with a sample-to-detector distance of 2.0 cm. Four scans were needed to cover the full width of the sample. The resulting volume was of size 2584 × 16606 × 16606 voxels, corresponding to a data volume of 2.6 TiB.

The eggshell was scanned with a 10× Mitutoyo objective and using a 100 µm-thick Lu Ce-doped lutetium aluminium garnet scintillator. The eggshell was scanned with 3 ms of exposure and 29 ms of latency per projection. The eggshell was used for Figs. 1 ▸ and 2 ▸, as a sample scan. The resulting volume was of size 2256 × 2176 × 2176 voxels, and a data volume of 39.8 GiB.

### 2.6. Performance testing

Benchmarking the presented reconstruction scheme is not trivial, as the bulk of the work is distributing tasks. For single computer benchmarking, the closest one can get is most likely *TomoPy*, which has already been benchmarked (Gürsoy *et al.*, 2014 ▸). The actual performance will vary greatly based on the cluster the reconstruction is running on and which rules that cluster applies to the users. For testing, we reconstructed the three volumes mentioned above, thrice each, using the MAX IV online cluster. Benchmarking was performed with 25 machines, each with 500 GiB of memory and 96 CPU threads. The MAX IV cluster holds 99 such machines and a further 28 machines with 350 GiB of memory and 64 CPU threads, making congestion another challenge for benchmarking. Note that the schema shown in Fig. 1 ▸ has some bottlenecks in between the primary steps. *i.e.* collection of the data cannot start until all chunks have been pre-processed; the reconstruction and post-processing cannot start until the data have been virtually collected; the post-reconstruction processes cannot start until the data have finished post-processing and reconstruction. At these points, another reconstruction can then come in and utilize free nodes. When running on 25 machines, multiple reconstructions often happen concurrently, until they can all finish a once. For the performance test, the reconstructions were run as a lone job.

Note that connectivity to the data storage is of particular importance. The MAX IV storage cluster uses SSD arrays with high-speed connections (>10 GB s^−1^); this makes it possible to read and write the data multiple times without adding much overhead compared with the computation time (Bell *et al.*, 2026 ▸). The largest dataset demonstrated here was 5.7 TiB in size. Across the reconstruction, reading and writing can easily add hours to the reconstruction time. Utilizing a slower server for storage connection will severely impede this part of the process.

### 2.7. Workflow at the beamline

At the beamline, the reconstruction parameters are found using an in-house GUI for *TomoPy* (https://github.com/in-kant/Reconstructor). This GUI produces a JSON-encoded file, a tomographic reconstruction information (.tori) file containing all required settings and values to perform the reconstruction. Another GUI will combine the .tori file, a scan file, and potentially a rotation centre to produce a task file. This task file is then read by a daemon running on the MAX IV server, which submits the reconstruction job.

## 3. Results and discussion

**Figure 4:**
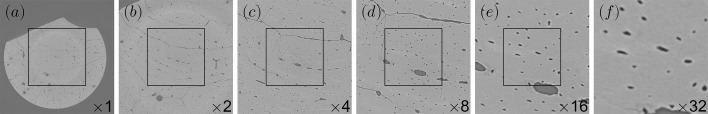
Reconstruction of ovine bone sample (*a*–*f*), shown with progressive zoom levels. The box on each figure marks the region shown in the next figure. The boxes in figures (*a*–*e*) have widths of 2.2 mm, 1.1 mm, 550 µm, 275 µm and 137.5 µm, respectively. The pixel size is 0.275 µm.

Slices of the large ovine bone dataset are shown in Fig. 4 ▸, with Figs. 4 ▸(*a*)–4(*f*) showing ovine bone. The figure illustrates how far you can zoom in while retaining a high level of detail, due to the high voxel density in the volume. The reconstruction method has no problem working with volumes of this size. Ideally, data like this would use full-field imaging with a large beam and a large high-resolution sensor. This is currently not possible at DanMAX due to the beam size of 1 mm. Thus, the data were collected using stitch projections. The samples were somewhat influenced by the high dose and high dose rate caused by 10^12^ photons s^−1^ mm^−2^ on the sample. The centre can be successfully reconstructed, as shown in Fig. 4 ▸(*f*). However, the regions further away from the rotation centre expanded/contracted enough so that the final reconstruction could not be used for, for example, quantification purposes. The optimizing of the scanning process is an ongoing work, together with the installation of a beam-expander at the DanMAX beamline to avoid the problem in the future (Gellert *et al.*, 2025 ▸).

The results of the performance testing are shown in Table 1 ▸. In all cases, phase retrieval using the Paganin method (Paganin *et al.*, 2002 ▸) has been performed. The reconstruction time scales with the dimensions of the volume and, especially for the largest dataset, the reconstruction time can be rather long. For the largest scan, reconstructions require 70 GiB of memory per slice during the computation. That makes it infeasible to reconstruct on most modern GPUs, and why these were all processed using CPUs only. As datasets are foreseen to continue to grow in size, this will likely continue to be a problem. The overlap of the subchunks for the pre-processing shown in Fig. 1 ▸ becomes a larger portion of the data loaded at larger data sizes, thus the processing time to larger datasets does not scale completely linearly with data size.

## 4. Conclusion

We have presented a new pipeline for reconstructing tomographic data. The algorithm is intended for future tera-voxel datasets expected to be routinely available with new medium-format CCD sensors. The pipeline leverages highly parallel computing to distribute jobs to utilize the available memory and compute resources. The framework is flexible and can be used with different backends for the tomographic reconstruction. Utilizing the current MAX IV cluster, we have demonstrated that this pipeline is highly scalable, reconstructing small, 40 GiB, volumes in 3 min, medium, 2.6 TiB, volumes in ∼1.5 h, and large, 5.7 TiB, volumes in 3.25 h.

The pipeline is currently in use for tomographic reconstruction at the DanMAX beamline at MAX IV on smaller datasets, but here we have shown that this scheme is scalable, and that using the MAX IV HPC cluster it can reconstruct tera-voxel volumes.

## Supplementary Material

Table S1. DOI: 10.1107/S1600577526004856/tv5088sup1.pdf

## Figures and Tables

**Table 1:** Results of the performance test Sample, data volume, file size and reconstruction time are shown. For the reconstruction time, the average of three reconstructions of the same dataset was used. A benchmark with 15 machines with 350 GiB memory and 64 CPU threads, but no IO and slurm times, is shown in Table S1 of the supporting information.

Sample	Egg shell	Mouse brain	Ovine bone
Raw data volume	2258 × 2256 × 2176	24002 × 2586 × 24609	25134 × 2584 × 16606
Reconstructed data volume	2256 × 2176 × 2176	2584 × 24608 × 24608	2584 × 16606 × 16606
Reconstructed file size	39.8 GiB	5.7 TiB	2.6 TiB
Reconstruction time	181 s	3 h 15 min 20 s	1 h 36 min 48 s
IO time (fraction)	77 s (42.4%)	32 min 27 s (16.6%)	21 min 12 s (21.9%)
Slurm time (fraction)	5 s (2.5%)	10 s (0.08%)	11 s (0.18%)
Raw data pixels processed per second	61.4 × 10^6^	130.3 × 10^6^	185.7 × 10^6^

## Data Availability

The code for this work is available at the MAX IV github, https://github.com/maxiv-science/reconBlazer, and at Zenodo, https://doi.org/10.5281/zenodo.19111509
