# Supplementary material for: Distributed computing for the reconstruction of multi-terabyte tomographic X-ray imaging datasets
Source: J Synchrotron Radiat. 2026 Jun 2;33(Pt 4):1103–9. doi: 10.1107/S1600577526004856 (PMC13344534; doi:10.1107/S1600577526004856)
Supplement: Supplementary file 1 [file s-33-01103-sup1.pdf]

JOURNAL OF SYNCHROTRON RADIATION

Volume 33 (2026)

Supporting information for article:

Distributed Computing for the Reconstruction of Multi-terabyte Tomographic X-ray Imaging Datasets

Thorbjørn Erik Køppen Christensen, Frederik Holm Gjørup, Mads Ry Vogel Jørgensen, Adrian Rodriguez-Palomo, Anders Bjorholm Dahl and Innokenty Kantor

The original benchmarking test did not include the breakdown of slurm/IO overhead. The MAX IV cluster was upgraded in the meantime, so the original benchmark is included here. The tests in Table S1 were run on 15 nodes with 350 GiB of memory and 64 CPU threads.

Table S1 Performance test

Results of performance test, showing sample, data volume, file size, and reconstruction time. For the reconstruction time, the average for three reconstructions of the same dataset was used.

Sample	Egg shell	Mouse brain	Ovine bone
Raw data volume	2258×2256×2176	24002×2586×24609	25134×2584×16606
Reconstructed data volume	2256×2176×2176	2584×24608×24608	2584×16606×16606
Reconstructed file size	39.8 GiB	5.7 TiB	2.6 TiB
Reconstruction time	179 s	12 H:16 M: 16 s	6 H: 25 M: 4 s
Raw data pixels processed/s	$61.9 \cdot 10^{6}$	$34.6 \cdot 10^{6}$	$46.7 \cdot 10^{6}$
